# The Perceived Job Performance of Child Welfare Workers During the COVID-19 Pandemic

**DOI:** 10.1007/s10615-022-00861-z

**Published:** 2023-01-20

**Authors:** Tamar Axelrad-Levy, Talia Meital Schwartz Tayri, Netta Achdut, Orly Sarid

**Affiliations:** 1 The Charlotte Jack Spitzer Department of Social Work, Ben-Gurion University of the Negev, Be’er Sheva, Israel; 2 Child psychiatry and the Center for Child Development and Mental Health, Jewish General Hospital, McGill University, 845 Sherbrooke St W, Montreal, Quebec H3A 0G4 Canada

**Keywords:** Child welfare service, Perceived job performance, Job environment, Perceived stress

## Abstract

While the evidence on the adverse impact of the COVID-19 pandemic on the well-being of frontline social workers is emerging, the research on the impact of the pandemic on their performance is scarce. The presented study explores how the relationship between work environment predictors and perceived stress explains the job performance of child welfare social workers during the pandemic using survey responses of 878 child welfare social workers. The findings revealed the mechanism through which environment predictors and perceived stress interacted in explaining job performance during a time of large-scale crisis. We found that C.W. social workers who experienced greater COVID-19-related service restrictions reported poorer job performance, that perceived stress disrupted workers’ supervision and autonomy, and that supervision and job autonomy protected C.W. social workers from the adverse effects of perceived stress and workload on their job performance. Conclusions included the importance of autonomy and supervision in mitigating job-related stressors and the need to adapt and enhance child welfare supervision during times of national crisis.

## Introduction

Since the outbreak of the COVID-19 pandemic, reports of child abuse and the number of emergency interventions in families known to child welfare services have risen dramatically in many countries (e.g., Arazi & Sabag, 2020; Campbell, 2020; Katz et al., 2021). Despite the increased caseloads, child welfare services operated under frequently modified social-distancing restrictions, which included repeated mandated lockdowns, self-quarantines (Tener et al., 2020; U.S. Department of Health and Human Services, 2022), staff shortages, and service cutbacks (e.g., Ashcroft et al., 2022; Katz et al., 2020; Marmor et al., 2021). Recent studies have documented the adverse effect of the pandemic on the social work workforce, and revealed that frontline social workers had experienced elevated stress levels (Banks et al., 2020; Schwartz-Tayri, 2022) because of both their own personal hardships and those of their clients (Holmes et al., 2021). While knowledge about the repercussions of the pandemic on the well-being of social workers working with children and families is emerging, empirical research on the job performance of social workers is still lacking. This article examines the relationship between work-environment predictors (service restrictions, workload, supervision, and job autonomy) and perceived stress in explaining the perceived job performance of child welfare (C.W.) social workers during the COVID-19 pandemic.

### Child Welfare Services During the Pandemic

The COVID-19 pandemic had a devastating impact on children at-risk. Studies around the world reported a significant increase in reports of suspected (e.g., Swedo et al., 2020) and confirmed (e.g., Sidpra et al., 2021) child abuse. In the U.S., the percentage of Emergency Department visits related to child abuse and neglect ending in hospitalization increased significantly among children under 18, from 2.1% in 2019 to 3.2% in 2020 (Swedo et al., 2020). In France, a report documented a 30% increase in children’s exposure to family violence; in Brazil, domestic violence reports’ estimates jumped 40–50%. Similar trends were observed in China and Italy (Campbell, 2020), Canada (Bucerius et al., 2021), and the United Kingdom (Katz et al., 2021).. In Israel, since the onset of the pandemic, intrafamilial sexual abuse increased by 41%, and children’s exposure to domestic violence increased by 16% compared to the same period in 2019 (Arazi & Sabag, 2020). The Israeli Ministry of Social Affairs reported a rise of 760% in domestic violence reports during the first phase of lockdown in March 2020 (Katz et al., 2021).

Emergency restrictions enforced in Israel at the outset of the pandemic included a complete shutdown of the market, including all frontline social welfare services and child protection (Katz et al., 2021). In mid-March 2020, some local C.W. agencies returned to work with limited capacity. Still, C.W. social workers worked mainly remotely, via phone calls and video chats, except for family emergencies (Schwartz-Tayri, 2022). Most child residential care facilities were closed, forcing children to return to unsafe environments and abusive family members, thereby escalating the children’s exposure to multiple risks (for review, see Sect. 3.3. COVID-19 responses in Katz et al., 2021). Many families and children under the surveillance of C.W. services did not have access to digital devices that would have allowed them to access services remotely (Mishna et al., 2021). The constant changes in social distancing regulations forced C.W. social workers to move back and forth between face-to-face services and digital platforms, which affected continuity and trust-building in their relationships with children and families (Baginsky & Manthorpe, 2021; Cook & Zschomler, 2020; Jones & Westlake, 2021). These disruptions in C.W. service provision occurred in England, South Africa, and Israel, among others (Baginsky & Manthorpe, 2021; Fouche et al., 2020; Levine et al., 2020; Rasool, 2020). Consequently, social workers in many countries faced new challenges, including limited face-to-face interaction with children and families, and the difficulty of engaging children in remote therapeutic clinical interventions (Ashcroft et al., 2022).

### Theoretical and Empirical Background for Explaining Social Workers’ Job Performance

Job performance is the degree to which C.W. social workers’ interventions and service procedures positively influence client clinical outcomes and are consistent with ethical and best practice standards (Martin & Kettner, 1996; Megivern et al., 2007), which are the core objectives of social work services with children and families (Yoo et al., 2007). According to Levin et al. (2022) *perceived* job performance is the extent to which social workers believe they fulfill the expectations associated with their role in the agency (Levin et al., 2022). In modern countries, such as the U.S. and Israel, child welfare services play a significant role in determining the outcomes of vulnerable children (Glisson et al., 2012). C.W. social workers are at the center of C.W. service delivery, as they guide the ways in which services are provided (Nelson & Steele, 2007) and form close relationships with children and families (Mayer et al., 2009). Therefore, their job performance is one of the most influential aspects of child welfare service quality. Still, studies that systematically investigate the perceived job performance of C.W. social workers are rare (Kim & Kao, 2014; Levin et al., 2022).

#### Determinants of Job Performance

Work-environment factors strongly influence the morale and job performance of workers. The job environment of C.W. social workers can elevate workers’ job satisfaction, commitment, and job attitudes (Glisson et al., 2012), as well as lead to better clinical and functional outcomes (e.g., Glisson & Green, 2006, 2011). Poor working environments are negatively associated with workers’ motivation and performance (Glisson et al., 2012). Constraints, such as heavy workloads, demands to work for long hours, and lack of autonomy at work, create pressure on workers and, therefore, adversely impact the quality of their performance (Bruggen, 2015; Johari et al., 2018) and their well-being (Ben-Ezra & Hamama-Raz, 2020). This is especially relevant to frontline welfare providers in times of large-scale crisis (McFadden et al., 2021).

The Job Demand-Resources (JD-R) model views one’s work environment as comprised of resources and demands that determine work-related outcomes (Bakker & Demerouti, 2017). Job demands, such as service restriction and workload, require workers to expend effort and energy. Therefore, high job demands can expose workers to elevated stress, and thereby impair work-related outcomes, such as work performance and personal well-being. Job resources are those physical, psychological, social, or organizational aspects of the job that provide possibilities for achievement and support by meeting psychological needs (e.g., autonomy, different forms of support), thereby increasing motivation and productivity (Bakker & Demerouti, 2017). According to the JD-R model, job resources can buffer workers against the negative effect of job demands on various work-related outcomes (Bakker & Demerouti, 2017). In this study, we examined the following demands and resources as determinants of social workers’ perceived job performance: workload and service restrictions as job demands, and job autonomy and professional supervision as job resources.

Workload is a mental construct that reflects the mental strain resulting from performing a task under specific environmental and operational conditions, coupled with the operator’s capacity to respond to those demands (Omolayo & Omole, 2013). Quantitative workload refers to the amount of work a person is asked to complete in a given time– “in the case of social workers, [workload] would cover pressures placed upon the individual social worker, such as unmanageable caseloads, time pressure, working with deadlines that are too tight or being asked to take too much responsibility” (Blomberg et al., 2015, p. 2019). From this perspective, workload imposes demands on the individual who may not have enough resources (e.g., time, professionals’ guidance) to overcome them (Spagnoli et al., 2020), may threaten workers’ well-being, and therefore is expected to hamper worker performance. We, therefore, expected that workloads placed on C.W. social workers during the onset of the pandemic would be associated with elevated personal stress and impede their job performance.

Service restrictions refer to large-scale (often nationwide) changes in the scope of staff, work hours, and programs available for service provision. Inherently, service restrictions reduce the resources available for the provision of services (Grootegoed & Smith, 2018; Ravalier, 2019) and increase the stress imposed on frontline social workers because of the need to provide services with a shortage of resources; they therefore predict adverse outcomes at the worker level (Siegrist et al., 2012). This mechanism suggests that service restrictions may indirectly impact C.W. social workers’ job performance by increasing stress. Pre- and post-pandemic studies have demonstrated that service restrictions associated with heavier workloads are linked to the social workers’ experiences of stress in their personal lives (Antonopoulou et al., 2017; Banks et al., 2020; Ben-Ezra & Hamama-Raz, 2020; Ellett et al., 2007; Griffiths et al., 2017; Morazes et al., 2009; Yehudai et al., 2020). Considering the reported impact of the pandemic on C.W. services worldwide (Katz et al., 2021), we expected that greater service restrictions experienced by C.W. social workers during the pandemic would be associated with a heavy workload and perceived stress, and would impede job performance.

Job autonomy refers to the extent to which a particular job can provide freedom, independence, and discretion to the individual in scheduling work and determining the procedures to implement (Johari et al., 2018). Autonomy leads to a critical psychological state whereby the workers feel responsible for the outcomes of their work (Hackman & Oldham, 1975). Therefore, job autonomy can motivate workers to perform better at work (Cascales Mira, 2021; Green & Mostafa, 2012), especially in relatively low-paid professions such as social work (Gallie et al., 2012; Kalleberg, 2011). Additionally, exercising greater autonomy may have enabled frontline workers to adapt their practice to the reported pandemic-related service restrictions and changes in C.W. service provision. Studies conducted among social workers empirically supported the link between autonomy and job performance (Abramovitz & Zelnick, 2015), as did studies of other health and social professionals, such as nurses (Hee et al., 2016) and teachers (Johari et al., 2018). In child welfare services, greater job autonomy was associated with positive job-related outcomes, such as decreased turnover intentions and lower burnout (Kim & Stoner, 2008). Based on this empirical linkage, we assumed that C.W. social workers who exercised greater autonomy would report lower perceived stress and increased job performance.

Professional supervision is embedded within the routine of social welfare services to support workers’ professional decision-making and acquisition of skills (Caspi & Reid, 2002). Therefore, good quality supervision is essential in facilitating social workers’ performance (Carpenter et al., 2012; Choi, 2017; Hunt et al., 2016; Mor-Barak et al., 2009; Revell & Burton, 2016). Supervision was also related to social workers’ well-being, management of stress (Gibson et al., 1989; Revell & Burton, 2016), job satisfaction, and professional efficacy (Choi, 2017), and retention (Carpenter et al., 2012; Chiller & Crisp, 2012). In child welfare services, poor supervision was associated with high burnout (General Accounting Office, 2003) and low retention (Chen & Scannapieco, 2010; Kim et al., 2011; Lizano & Mor-Barak, 2012). Therefore, we assumed that supervision would be positively associated with perceived job performance during the COVID-19 pandemic, and would mitigate the negative effects of perceived stress and workload on job performance.

Previous studies have shown that social workers’ professional seniority is related to outcomes at the individual level. More specifically, seniority was found to positively associate with the belief in self-accomplishment (Levine et al., 2020), job satisfaction (Godas Otero et al., 2022; Jiang et al., 2019), and ethical conflicts (Zychlinski et al., 2020); and to negatively associate with social workers’ burnout (Tartakovsky, 2016), psychological distress (Kagan & Itzick, 2019), and intentions of leaving the profession (Itzick & Kagan, 2017). Still, studies examining the link between social workers’ seniority and perceived job performance are scarce, as most studies have considered age instead (Zychlinski et al., 2020). Therefore, we suggest that seniority should be considered when attempting to explain C.W. social workers’ perceived job performance.

### The Current Study

With the frequent changes in social distancing regulations, staff shortages and heavy workloads, social workers had to frequently adapt service provision during the first outbreaks of the pandemic(e.g., Ashcroft et al., 2022; Katz et al., 2020; Marmor et al., 2021). Social workers reported personal stress (Ben-Ezra & Hamama-Raz, 2020; Holmes et al., 2021) and professional concerns about caring for young children at risk (Ashcroft et al., 2022). Considering the impact of COVID-19 on the work environment of social workers, we assumed that C.W. social workers needed to constantly adapt service provision. This included reaching out to children and families with limited face-to-face access in the context of frequent changes in social workers’ working conditions and increased personal stress. Consequently, we assumed that social workers’ job performance was hampered (Priolo Filho et al., 2020; Seddighi et al., 2021). This study empirically assessed the perceived job performance of child welfare (C.W.) social workers during the first waves of the pandemic in Israel.

Integrating the JD-R model theoretical mechanism with empirical evidence, we assumed that, while demands in the forms of service restrictions and workload would increase the perceived stress of C.W. social workers and adversely impact their job performance, supervision and job autonomy would mitigate such impacts. More specifically, we hypothesized that: (1) Workload and perceived stress would mediate the relationship between service restrictions and perceived job performance; (2) supervision and job autonomy would mediate the relationship between stress and perceived job performance; and (3) supervision and autonomy would meditate the relationship between workload and perceived job performance.

## Methods

### Participants and Data Collection

We collected the data during the first two waves of COVID-19 (July to August 2020) using an online Qualtrics software survey. The Israeli National Association of Social Workers sent the invitation to participate in the survey to all licensed Israeli social workers registered in the association. From a list of 1664 social workers in child welfare services, 878 completed the survey, making a response rate of 52.76%. Participants’ ages ranged from 22 to 50 years (M = 32.18 years, SD = 6.8); 92.1% were women. Among participants, 93.1% described themselves as heterosexual, 4.5% as gay or lesbian, and 0.5% as questioning. As for family status, 78% were married or living with a partner, 12.7% were single, and 9.4% were separated or divorced. Participants’ length of experience in children and youth services ranged from one to 36 years (M = 7.33 years, SD = 6.47). At the time of the study, 79% of the participants worked in the public sector, including local child protection agencies; 19% in the non-profit sector; and 1.9% in for-profit sector agencies.

### Ethical Statement

The study adhered to the ethical guidelines of the authors’ university and was approved by the Institutional Review Board (Approval number: #12,047). The Qualtrics IP details collection option was disabled to ensure maximum anonymity of participants. Informed consent was required before participants were directed to the survey.

### Measurement and Instruments

#### Perceived Job Performance

Perceived job performance was assessed using a 13-item scale developed by Abramovitz and Zelnick (2018) in social work services research. Participants were asked to indicate whether each dimension described 1 = does not happen here, 2 = not a problem, 3 = minor problem, or 4 = major problem. These dimensions included, for example, “Not enough time to adequately assess the needs of children we serve.” Scores were calculated as the mean of all items. We reversed the scale so that higher scores signified better service performance. Cronbach’s alpha = 0.83.

#### Supervision

Supervision was assessed using a 7-item scale developed by Abramovitz and Zelnick (2018) in social work services research. Participants were asked to evaluate the quality of the supervision they received as part of their work in the agency. Items included, for example, “We are not offered enough supervision to do my job effectively” or “Training topics are not useful in my job.” Participants were asked to rate each statement on a 4-rank Likert scale: 1 = doesn’t happen here, 2 = not a problem, 3 = minor problem, 4 = major problem. Scores were calculated as the mean of all items. We reversed the scale so that higher scores signified better supervision. Cronbach’s alpha = 0.65.

#### Job Autonomy

We used a single item to measure global autonomy (Abramovitz & Zelnick, 2018). Participants were asked, “How much control do you feel you have over your work?” and gave their response on a 1–4 scale: 1 = not at all, to 4 = very much. A higher score signified greater work autonomy.

#### Service Restrictions

Service restrictions were assessed using a 5-item scale designed for the study. It measured negative changes in social workers’ work settings that were implemented due to the COVID-19 outbreak and social distancing requirements. Participants were asked to indicate (yes, no, or not applicable) whether any of the following occurred at the agency in which they worked in response to the pandemic outbreak: (1) service closure; (2) program closure; (3) staff cutbacks or shortage of staff; (4) state or city budget cuts; and (5) reduction of work scope. Scores were calculated as the percentages of items answered as “yes” from all of the items, ranging from 0–100 percent, with higher scores signifying greater service restrictions. Cronbach’s alpha was 0.72.

#### Perceived Stress

Perceived stress was assessed using the 10-item Perceived Stress Scale (Cohen et al., 1983). Participants rated on a 4-point Likert scale, ranging from 0 (never) to 4 (very often), how often in the previous month they had experienced certain stressful situations. For example, “In the last month, how often have you felt that you were unable to control the important things in your life?” Scores were calculated as the mean of all items, with higher scores signifying higher perceived stress. Cronbach’s alpha was 0.69.

#### Workload

The workload was assessed using four questions developed by Abramovitz and Zelnick (2018) in social work services research. Participants were asked to indicate whether each item reflected a phenomenon that: (1) does not happen in the agency, (2) not a problem, (3) a minor problem, or (4) a major problem. For example, “Can’t complete my work during normal work hours (e.g., take work home, stay late, work at lunch)” and “Having to work too fast.” Scores were calculated as the mean of all items, with higher scores signifying greater workload. Cronbach’s alpha was 0.84.

Background and professional characteristics included age, gender, family status, sexual orientation, ethnicity, and seniority in child and youth practice (years of tenure).

### Statistical Analysis

We used SPSS 26 to conduct all data analysis. The rate of missing data ranged from 1.5% to 19%. We employed Little’s MCAR test to decide whether the data had missing values in a random pattern (Collins et al., 2001). The analysis revealed that the data were missing completely at random: chi-square (150) = 140.231, p = 0.705. We performed maximum likelihood using SPSS 26 to recover for missing values, making it possible to use the entire data set. In the next analysis stage, we calculated Pearson correlations between background variables (age and seniority level in child and youth practice) and the five main study variables.

As seniority in child and youth practice significantly correlated with the study outcome variable, perceived service performance (r = 0.08, p < 0.001), it was added to regression analysis. A three-step hierarchical linear regression analysis was employed to determine the independent variables’ contribution to the outcome, perceived job performance. Finally, we used the PROCESS procedure in SPSS macro to test serial mediations (Hayes, 2013; Hayes et al., 2011). This analysis examines all possible direct and indirect effects through the mediating variables and allows us to elucidate the explanatory relationships between the outcome and predictors and reveal under which circumstances they are valid (Hayes & Rockwood, 2020). The analysis uses a bootstrapping method with 1000 bootstrap resamples and generates an estimate of all indirect effects, including a 95% confidence interval (Hayes, 2013). When zero is not in the 95% confidence interval, one can conclude that the indirect effect is significantly different from zero at p < 0.05 (two-tailed).

## Results

### Descriptive Analysis and Intercorrelations

Table 1 shows the descriptive statistics and intercorrelations between the study variables. Among background characteristics, only seniority correlated significantly with job performance (r = 0.08, p < 0.001). Therefore, seniority was incorporated in regression analysis.

**Table 1:** Descriptive statistics and intercorrelations among variables

	Mean (S.D.)	Scale	1	2	3	4	5	6	7
1. Preceived job performance	2.07 (.56)	1–4	1						
2. Service restrictions	42% (34%)	0–100%	−.36**	1					
3. Workload	2.28 (76)	1–4	− .56**	.28**	1				
4. Seniority	15.56 (10.35)	–	.08**	− .01	− .02	1			
5. Job autonomy	1.78 (.96)	1–4	.52**	.24**	.67**	− .02	1		
6. Supervision	2.24 (.52)	1–4	.45**	− .37**	− .44**	− .01	− .01	1	
7. Perceived stress	3.20 (.41)	0–4	− .28**	.17**	.37**	− .05	− .05	.25**	1

Pearson’s correlation coefficients were presented. *p < 0 0.05; **p < 0.01

### Regression Analysis

To determine whether including the independent variables and covariates in the current analyses, especially between job autonomy and workload, was adequate, we assessed for multicollinearity and examined the variance inflation factors (VIFs) for the study variables of interest. Findings indicated that all were within the acceptable range (all VIFs were smaller than 2, indicating that multicollinearity was not a problem in our analyses.

First, we employed regression analysis. The findings showed that service restrictions, workloads, and perceived stress were associated with decreased perceived job performance, while supervision and job autonomy were associated with increased perceived job performance (Table 2). The study variables explained 44% of the variance in job performance. Also, higher levels of seniority in child and youth practice were associated with increased perceived job performance.

**Table 2:** Summary of a three-step hierarchical multiple regression analysis for predicting perceived job performance

	b	S.E. b	β	R^2^
*Model 1*				.08
Seniority	.004	.002	.07*	
Perceived stress	− .38	.04	− .28***	
*Model 2*				.37
Seniority	.005	.001	.08*	
Perceived stress	− .16	.01	− .35***	
Supervision	.31	.03	.29***	
Job autonomy	.20	.01	.35***	
*Model 3*				.44
Seniority	.004	.001	.07*	
Perceived stress	.064	.003	− .04	
Supervision	.22	.034	.21***	
Job autonomy	.10	.021	.17***	
Service restrictions	− .002	.000	− .15***	
Workload	− .218	.027	− .29***	

*β* = standardized beta, SE b standard error of b. * p < .05. ** p < .001

### Revealing the Mediating Mechanisms in Explaining Service Performance

**Fig. 1:**
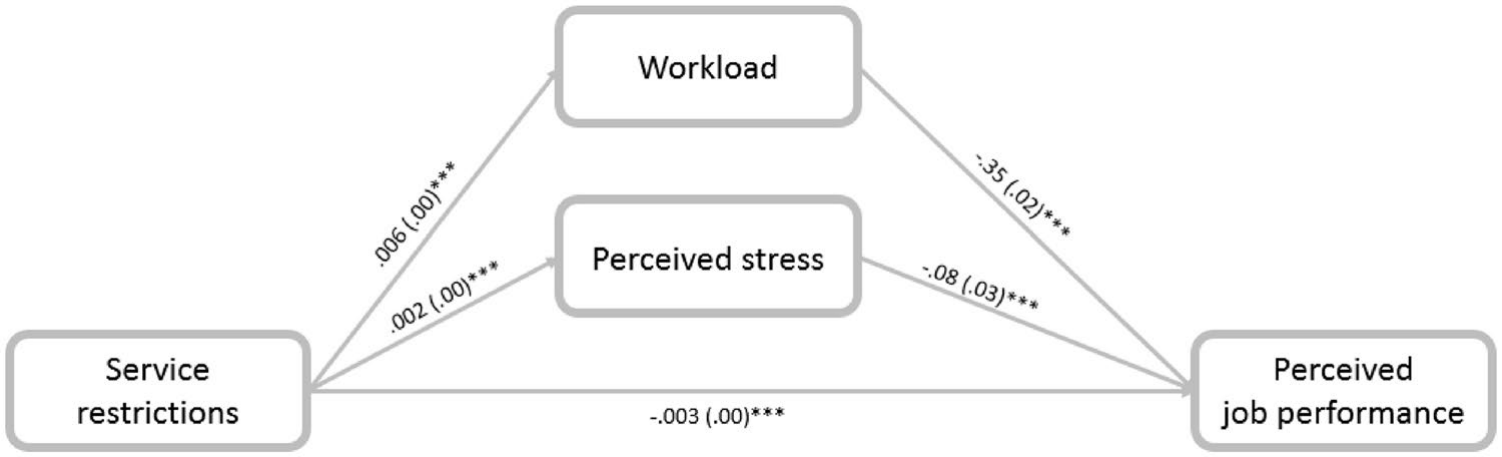
Mediation of the association between service restrictions and perceived job performance through workload and perceived stress

We conducted mediation analyses to examine the mitigation effects using the PROCESS procedure in SPSS 26. Findings revealed that C.W. social workers who experienced greater service restrictions reported greater workloads (− 0.0023; CI: − 0.0028, − 0.0017), which led to decreased perceived job performance (Fig. 1). The indirect effect of service restrictions on perceived job performance through perceived stress was insignificant (− 0.0002; CI: − 0.0004, 0.0000); in other words, while workload was a significant mediator in the relationship between service restriction and perceived job performance, perceived stress was not a significant mediator. Therefore, hypothesis (1) was only partially confirmed.

**Fig. 2:**
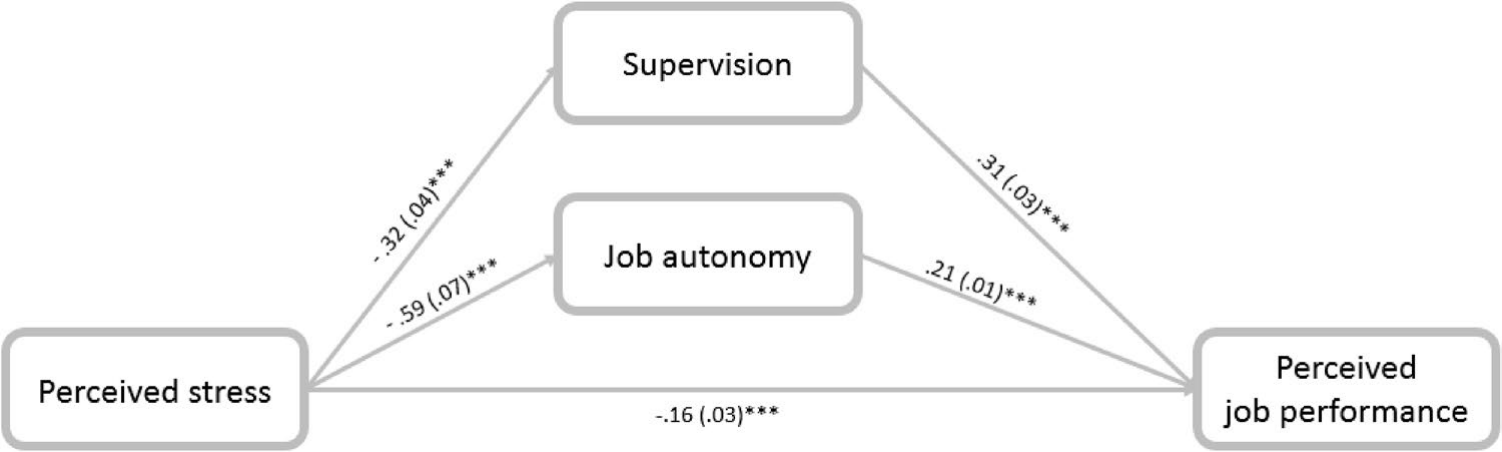
Mediation of the association between perceived stress and perceived perceived job performance through supervision and autonomy

Perceived stress was associated with perceived job performance directly and indirectly through the mediation of supervision (− 0.0981; CI: − 0.1357, − 0.0643) and job autonomy (− 0.13; CI: − 0.1638, − 0.1735), confirming hypothesis (2). C.W. social workers who were more stressed reported poorer supervision and less autonomy, which was later associated with perceived job performance (Fig. 2).

**Fig. 3:**
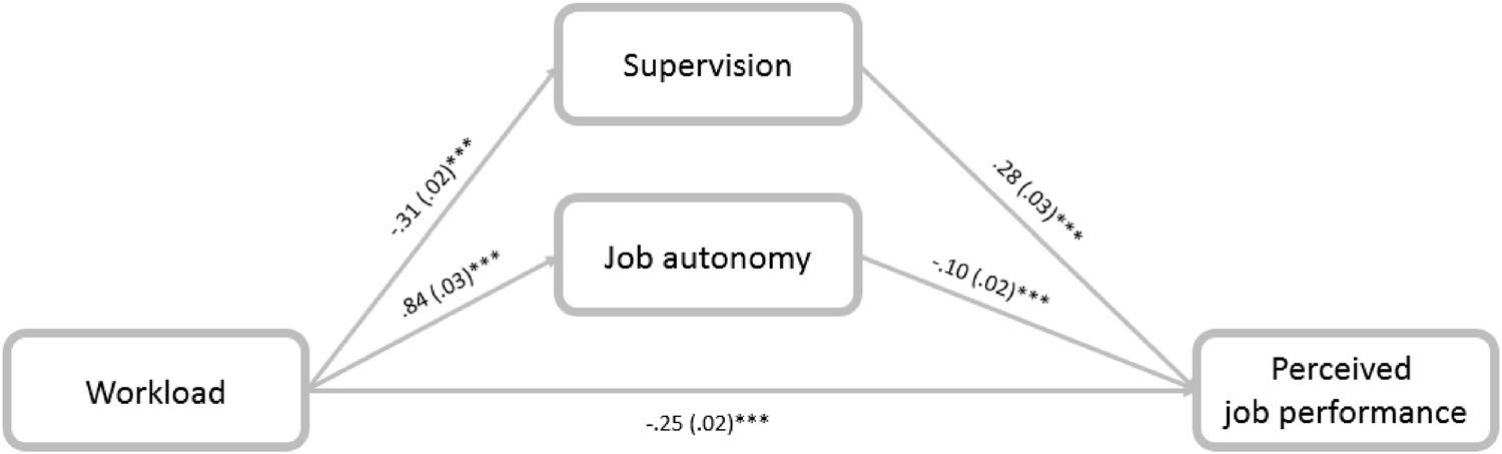
Mediation of the association between workload and perceived job performance through supervision and autonomy

Workload was associated with job performance directly and indirectly through the mediation of supervision (− 0.0864; CI: − 0.1141, − 0.0604) and job autonomy (− 0.08; CI: − 0.1214, − 0.0465), confirming hypothesis (3). C.W. social workers who dealt with greater workloads reported poorer supervision and greater job autonomy, which was later associated with perceived performance (Fig. 3).

## Discussion

This study used the JD-R theoretical model to investigate *how* work-environment predictors interacted in explaining the job performance of C.W. social workers during the first two waves of the COVID-19 pandemic. We found that C.W. social workers who experienced greater COVID-19-related service restrictions reported poorer perceived job performance and that perceived stress disrupted workers’ supervision and autonomy. We also discovered that supervision and job autonomy protected C.W. social workers from the adverse effects of perceived stress and workload on their job performance. Our findings corroborate previous evidence on the adverse impact of the pandemic on the well-being of frontline service providers (e.g., Ben-Ezra & Hamama-Raz, 2020; Elyashar et al., 2021; Greene et al., 2020; Martínez-López et al., 2021; Williamson et al., 2020), and expand the knowledge on how this adverse impact impeded frontline workers from performing adequately while serving the most vulnerable populations of children.

Our findings expand the JD-R framework by revealing how service restrictions, as contextual crisis circumstances, are linked with in-job resources and demands determining C.W. social workers’ performance. Studies that sought to examine the determinants of worker-level outcomes, such as work engagement, burnout, and turnover, in child services settings have focused on worker- and organizational-level predictors and revealed the relationships between the worker and the organizational environment to explain workers’ outcomes (e.g., Hussein, 2018; Lee et al., 2019; Lizano & Mor-Barak, 2012; Olaniyan et al., 2022). This study adds to the existing literature on the role of service restrictions, as an organizational feature derived from the broader context, in creating a stress sequence reaction that eventually affects the quality of service provided to the populations that most needed them during the crisis. Like previous global crises, the COVID-19 crisis prompted governments to enforce immediate social distancing regulations, fettering the ability of local child welfare services to access children at risk and compelling them to focus on emergencies only (Miller & Hokenstad, 2014). Policymakers in Israel did not adequately assess the possible harm that closing child welfare services would have on at-risk children’s adversity, as shown by the rapid rise in domestic violence reports.

Our findings show that the link between the broader policy context and C.W. social workers’ outcomes can be explained by the interaction of work-environment stressors with protective factors. During the first waves of the pandemic, C.W. social workers who experienced service restrictions reported job stressors–workload and perceived stress. In contrast, supervision and job autonomy buffered the adverse effect of job stressors on workers’ perceived job performance. High caseloads are part of the routine of C.W. services (Antonopoulou et al., 2017; Baldschun et al., 2016; Tufford et al., 2021). Still, the mechanism that linked COVID-19-related service restrictions with increased workload and decreased perceived job performance emphasizes that the workload created in the context of limited service provision had exposed C.W. social workers to environmental demands to which they could not adapt. Although supervision and autonomy significantly buffered the adverse effects of perceived stress and workload on perceived job performance, these maintained their direct negative effect on perceived job performance. Moreover, findings showed that perceived stress hampered performance by weakening social workers’ sense of autonomy and their ability to use the supervisory relationship as a coping resource.

Altogether, the mechanism demonstrated supports the wear-and-tear stress-related explanation (McEwen, 2004). A career in C.W. services involves creating close relationships with young victims of trauma (Littlechild et al., 2016), constantly responding to family crises, and managing high caseloads and conflicting child-family-authority processes (Boyas et al., 2013; O’Donnell & Kirkner, 2009; Tufford et al., 2021). Hence, the cumulative effect of C.W. social workers’ daily exposure to elevated stressors prior to the outbreak of the pandemic (Lizano & Mor-Barak, 2012; Maddock, 2022; Travis et al., 2016), along with the acute increase in stressors during its first waves (Katz et al., 2021) operated as persistent, prolonged stress exposure, in which resources (autonomy and supervision) were not enough to protect C.W. social workers from decreased perceived performance.

The protective role of supervision and autonomy shows that processes embedded within the C.W. service routine can—at least to an extent—compensate for demands created by circumstances external to the agency. Supervision partially protected workers’ performance from the impact of workload and personal stress. While research conducted prior to the pandemic confirmed that supervision and related support resources are positively related to C.W. social workers’ outcomes (Hamama, 2012; Lei et al., 2019; Lizano & Mor Barak, 2012; Tufford et al., 2021), our findings demonstrate that supervision is crucial in enhancing workers’ performance in a time of crisis. Supervision was a significant source of support, offering workers the guidance to face the unfamiliar work circumstances brought about by the pandemic crisis. While autonomy protected workers from the effect of perceived stress on their performance, it decreased their performance when associated with elevated workloads. Taken all together, we suggest that workers who experienced greater workloads were forced to exercise greater autonomy, and may therefore have had to deal with demands alone, which eventually jeopardized their performance. The differences in the roles of supervision and autonomy highlight that, in a large-scale crisis, workers may benefit from greater supervision as it can serve as a source of social support and buffer the toll of job demands (Antonopoulou et al., 2017; Astvik & Melin, 2013; Kim & Lee, 2009; Kim, 2011). At the same time, greater autonomy may place greater responsibilities on workers, who may then have to meet them with insufficient resources and higher demands.

Finally, mapping the restrictions imposed in the first waves of the pandemic raises serious questions concerning policymakers’ decision to immediately shut down all welfare services in Israel, including those services providing assistance to marginalized groups, such as women and children experiencing domestic violence (Katz et al., 2020). The history of the welfare system in Israel shows that, in times of national crisis, welfare services and social spending for children at-risk have been the first sectors to be subjected to austerity measures (Maron, 2021), partially due to the limited political power of those receiving such assistance (Binder, 2013), and, related to this, the low political power of social workers compared to that of workers in other sectors. It is possible that policymakers did not adequately consider the potential harm to populations at risk of closing welfare services, as initially the rights of these populations were not recognized by policymakers, who did not consider the receipt of welfare assistance as essential, in contrast to medical assistance, which was not restricted in any form or at any phase during the pandemic.

### Limitations

While this study provides significant evidence about the perceived job performance of C.W. social workers during the COVID-19 pandemic in Israel, several limitations should be considered. First, in this study, we employed cross-sectional methods on data collected following the first six-week lockdown. As such, it does not include data on the study’s main variables from before the pandemic outbreak. Second, we could not compare the levels of C.W. job performance found in this study with previous studies, as the research on the job performance of C.W. social workers in Israel is scarce. Hence, we could not conclude that circumstances created by the pandemic had a de facto role in shaping the relationship between the predictors in explaining job performance. Third, this research was conducted in Israel, where so far, social distancing measures have been stricter than those in other western countries (ECDPC, 2020). For example, the onset of the pandemic resulted in an early and complete shutdown of public services, excluding emergency services (Kondratjeva et al., 2021). Therefore, applying the present findings to C.W. social workers outside of Israel should be considered with caution. As a result of these limitations, child welfare research should seek to expand the knowledge on services performance using longitudinal examination and cross-countries comparisons to investigate the relationship between contextual, organizational, and personal predictors of C.W. social workers’ performance during large-scale crises. Fourth, the sample might also be skewed due to participants’ self-selection. It is possible that participants who completed the survey were more eager to express their challenges and thankful for the opportunity to have their voices heard than those who did not complete it. Additionally, all measures in the study, including job performance, are perceived measures, as we used self-reported measures, which risk social-desirability bias and therefore do not represent objectivity.

### Implications for Social Work Practice

The vast and rapid spread of the COVID-19 virus tested governments’ capacity to respond to the most vulnerable populations of children affected by the crisis brought about by the pandemic. The pandemic-related service restrictions discussed here provide evidence suggesting that the Israeli government’s social policy responses to the emergency caused by the pandemic were insufficient. Further, our explanatory model demonstrates that contextual, organizational, and personal level factors are essential in explaining the performance of C.W. social workers in times of large-scale emergencies. Therefore, in terms of national policy, the model highlights the need to create a child welfare multi-authority unique nationwide plan aimed at fostering the capacity of child and family welfare services to respond to the needs of the at-risk population quickly, widely, and with the proper tools. Additionally, welfare service managers should enhance C.W. services crisis-preparedness by adopting organizational-, self-, and group-care, and by supporting tools to foster professional resilience (Maddock, 2022). Embedding these tools in the routine of child welfare services can mitigate the likelihood of frontline C. W. workers experiencing negative psychological effects during times of emergency (Cabiati, 2021; Kranke et al., 2020).

The clinical implications of our findings for social workers’ practice and enhanced job performance can be translated into organizational and individual practice recommendations. Previous studies have shown that teaching and practicing cognitive-behavioral and mindfulness techniques with social work professionals reduced perceived stress (Sarid et al., 2010) and enhanced coping and well-being (Maricuţoiu et al. 2016; Slutsky, et al, 2019). On the organizational level, two pathways need to be noted. First, reducing stress and enhancing coping are needed when social workers are facing the detrimental effect of stress-related situations. This is relevant not only to social workers working with children who have suffered deprivation, but, also, to professionals working with individuals who have undergone traumatic events and suffering. Therefore, workplaces, especially those whose workers are prone to constant stress, are encouraged to supervise their employees and provide them with the option of learning and practicing various ways of reducing personal distress. On the individual level, learning by itself is not sufficient to reduce distress and improved mood states. It is the constant daily practice of the learned techniques that assists in mood regulation. Here, the organizations have an important role in tutoring the employees to adhere daily to self-soothing techniques. This might take the form of facilitating practice on a fixed schedule or through providing designated breaks. Dividing job demands in times of extreme stress, such as the COVID pandemic, can be done as a group to mitigate the loneliness of individuals segregated in their homes. We recommend that welfare agencies use digital platforms to promote a sense of shared responsibility and communication, in order to regulate individual distress by teaching and learning new coping strategies.
